# Comment on Alharbi et al. Adoption of Health Mobile Apps during the COVID-19 Lockdown: A Health Belief Model Approach. *Int. J. Environ. Res. Public Health* 2022, *19*, 4179

**DOI:** 10.3390/ijerph192416846

**Published:** 2022-12-15

**Authors:** Chinonyelum Nwosu, Kenneth D. Ward

**Affiliations:** Division of Social and Behavioral Science, School of Public Health, University of Memphis, Memphis, TN 38152, USA

Alharbi and colleagues’ article, “Adoption of health mobile apps during the COVID-19 lockdown: a Health Belief Model approach”, was interesting, well-written, and informative [1]. The background information on the handling of the COVID-19 pandemic was informative for other countries that did not have as successful of a response as Saudi Arabia. The development, distribution, and use of the mobile health apps prior to the pandemic and the foundational infrastructure that they were able to provide during the pandemic were impressive. The topic of this article is very timely and is a promising area of study.

We would like to comment on two issues that may help future studies on this important topic. The authors chose not to evaluate perceived susceptibility and severity as potential determinants of intended app use, although these are critically important constructs in the health belief model (HBM) as indices of threat. If an individual does not perceive an issue to be threatening, there is no motivation to consider the benefits and barriers to engaging in preventive behavior. The rationale for ignoring severity and susceptibility was that a similar study found that these constructs were not associated with intent to use a contact tracing app for COVID-19 [2]. However, Walrave et al.’s study was conducted in Belgium very early on in the pandemic, 17–19 April 2020, several months before the first major global surge of COVID-19 cases in November, 2020, when the 7-day rolling count of new COVID-19 cases in Belgium was about 116 cases/million, far lower than its eventual peak of about 4381 cases/million (https://ourworldindata.org/covid-cases, accessed on 8 May 2022). Thus, the perceived threat may have still been relatively minimal, explaining the lack of association with the intention to use the app. Since then, the perceived threat and susceptibility likely increased considerably and may have become important predictors of app use given the pandemic’s enormous sweep and deadly consequences. As such, future studies should evaluate all HBM constructs. Additionally, a wider age range should be assessed than the 18–30-year-olds in this study, since age is an important factor in many HBM constructs related to COVID-19-related app use, including the perceived threat and self-efficacy [3].

One other issue is that even if all constructs were assessed, HBM may not be the most useful model since it limits itself to intrapersonal-level cognitive determinants. App use, however, occurs in the context of wider interpersonal, community, and societal inputs. A person’s app use can be influenced by or affect their environment and social network. For instance, apps that are designed to increase physical activity but do not take into consideration external social and environmental factors can have negative side effects or exclude groups [4]. One potentially useful framework would be the diffusion of innovations [5]. DOI is a theory that focuses on relative advantage, simplicity, compatibility, observability, and trialability to predict how well an idea or new technology will be adopted by a population. DOI has been used successfully to develop and implement a variety of health behavior interventions (e.g., [6,7]) These constructs help to identify the key aspects of both the technology and its dissemination that help facilitate adoption. DOI could perhaps identify why the apps were not well adopted, even though access was not a barrier, by identifying whether the apps are too complicated, are not perceived to be useful, or are not compatible with the lifestyle of this population. During the pandemic, many services, including healthcare and education, were forced to change rapidly, and DOI was able to predict whether these changes to virtual services were successfully adopted [8]. Additionally, DOI was able to predict the intention to receive the COVID-19 vaccine in China, using social media as a communication channel [3].

Future studies exploring the adoption of a mobile health app during a pandemic or vaccination uptake for a novel virus can use DOI as a framework. The four main constructs, innovation, communication channels, time, and social system are ideal for understanding the way a new behavior and/or technology can be implemented and disseminated. A future study to encourage vaccination through the use of a mobile app could explore the innovation by measuring the perceived relative advantage of the vaccine and the compatibility, complexity, and trialability of the app, while using the app as a communication channel to disseminate information about the vaccine, measuring the time it takes to process the decision about the innovation, and how any changes in social norms affects the innovation’s adoption curve.

This study is a useful contribution to the literature, and we hope that our suggestions will help future studies test theory-based approaches to bolster mobile app use for COVID-19 and other public health problems.

## Data Availability

Not applicable.
